# Transcriptomics-driven lipidomics (TDL) identifies the microbiome-regulated targets of ileal lipid metabolism

**DOI:** 10.1038/s41540-017-0033-0

**Published:** 2017-11-07

**Authors:** Anirikh Chakrabarti, Mathieu Membrez, Delphine Morin-Rivron, Jay Siddharth, Chieh Jason Chou, Hugues Henry, Stephen Bruce, Sylviane Metairon, Frederic Raymond, Bertrand Betrisey, Carole Loyer, Scott J. Parkinson, Mojgan Masoodi

**Affiliations:** 1 Nestlé Institute of Health Sciences SA, Campus EPFL, Quartier de l’Innovation, Bâtiment H, 1015 Lausanne, Switzerland; 2 Service de biomédecine (BIO), Quartier UNIL-CHUV, Rue du Bugnon 46, CH-1011 Lausanne, Switzerland

## Abstract

The gut microbiome and lipid metabolism are both recognized as essential components in the maintenance of metabolic health. The mechanisms involved are multifactorial and (especially for microbiome) poorly defined. A strategic approach to investigate the complexity of the microbial influence on lipid metabolism would facilitate determination of relevant molecular mechanisms for microbiome-targeted therapeutics. *E. coli* is associated with obesity and metabolic syndrome and we used this association in conjunction with gnotobiotic models to investigate the impact of *E. coli* on lipid metabolism. To address the complexities of the integration of the microbiome and lipid metabolism, we developed transcriptomics-driven lipidomics (TDL) to predict the impact of *E. coli* colonization on lipid metabolism and established mediators of inflammation and insulin resistance including arachidonic acid metabolism, alterations in bile acids and dietary lipid absorption. A microbiome-related therapeutic approach targeting these mechanisms may therefore provide a therapeutic avenue supporting maintenance of metabolic health.

## Integrative Lipidomics: Microbiome regulation of lipid metabolism

Microbes multifactorially impact host lipid metabolism bearing a significant impact in health and disease. A team led by Mojgan Masoodi and Scott Parkinson at Nestlé Institute of Health Sciences (NIHS) developed an integrative data driven approach for predictive lipidomics investigations of host-microbial impacts on lipid metabolism. Results of *in-vivo* studies with germ-free mice inoculated with *E. coli* and *in-vitro* studies demonstrated the multifactorial nature of the impact of *E. coli* on arachidonic acid metabolism in the ileum and altered host inflammation and lipid absorption. The findings provide insights into understanding the host-microbiome interactions and identifying microbiome-related solutions for maintaining health and tackling disease. The systems approach presented is applicable to investigate broad range of microbiome dependent and independent alterations in host lipid metabolism.

## Introduction

The gut microbiome is now recognized as an important factor in pathogenesis of metabolic diseases and a target for therapeutic intervention to maintain and improve health.^1–3^ Several studies indicate that alteration of gut microbiota may play a key role in development of diseases associated with altered lipid metabolism.^4–7^ Technical developments in lipid characterization^8–11^ and database curation^12–14^ have facilitated the study of lipid metabolism. However, our knowledge of the molecular mechanisms underlying microbiome regulation of host lipid metabolism is limited and hampered by the complex nature and prevailing technical limitations within the microbiome and lipidomics fields.

The microbiome has been promoted as a potential target to regulate lipid metabolism and metabolic function. One approach to bring this to fruition is the use of predictive models as a method to reduce the “search space” to focus on pathways with the best chance of success in conjunction with legacy knowledge. In addition, recent advances in the annotation of databases (organism level databases like Reactome^15,16^ and lipid-related databases like LIPID MAPS^13^) and data integration from technical advances in other fields like transcriptomics, proteomics, metabolomics (and others) could provide an integrated view to identify specific intervention points and develop testable hypotheses. Another valuable tool available for investigating complex, highly interconnected biochemical transformations is genome-scale metabolic model (GEM)^17,18^ which can elucidate metabolic genotype–phenotype relationships within lipid metabolism. These have been used to make systems biology models of sphingolipid metabolism^19^ to analyze differences in adipose tissue physiologies,^20^ to study aberrant lipid metabolism in prostate cancer^21^ and for blood analysis in type 2 diabetes mellitus.^22^ All of these approaches have individual strengths and weaknesses; however, an integrated approach combining previous knowledge, curated databases and metabolic modeling have not been considered, especially in the context of lipid metabolism.

**Fig. 1:**
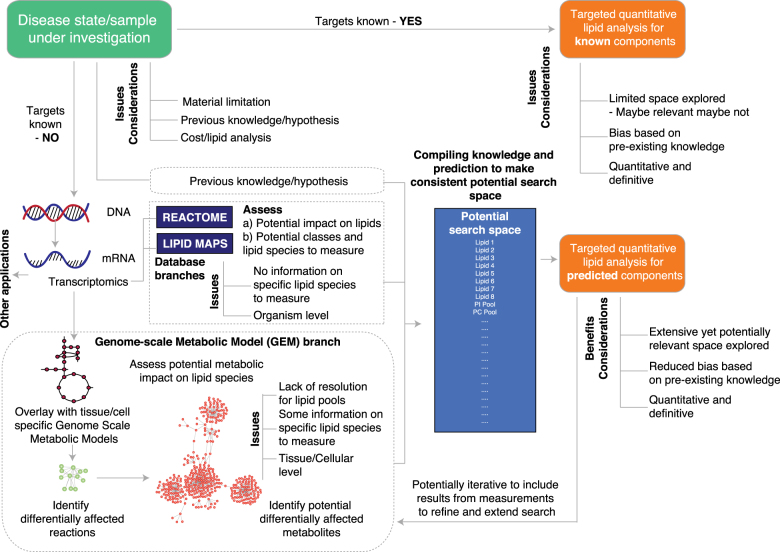
Transcription-driven lipidomics (TDL) strategy and its implications. TDL is an integrated strategy for hypothesis driven lipidomics survey coupling transcriptomics and combination of legacy knowledge, lipid databases, pathway databases and tissue specific genome-scale metabolic models to hypothesize predictions of potentially altered lipid metabolism (both at the level of specific lipid species and pathways) in health and disease

In order to identify key pathways involved in microbiome regulation of lipid metabolism we took advantage of gnotobiotic preclinical models and an *Escherichia. coli* (*E. coli*) strain isolated from an obesity mouse model (Ob/Ob). Analysis of mRNA levels in the ileum were put into context of lipid metabolism using an integrated approach considering legacy knowledge, lipid databases (e.g., LIPID MAPS^13^), pathway databases (Reactome^15,16^) and tissue-specific GEMs^23–26^ (further referred to as TDL (transcriptomics-driven lipidomics)) to predict likely changes in lipid metabolism in response to *E. coli* colonization (Fig. 1). Using TDL we predicted and demonstrated how *E. coli* colonization drives an increase in arachidonic acid metabolites and a decrease in components of glycerophospholipid metabolism via bacterial invasion leading to host inflammation, altered bile acid metabolism, and altered dietary lipid absorption.

## Results

### Preclinical model strategy to integrate microbiome and lipid metabolism

**Fig. 2:**
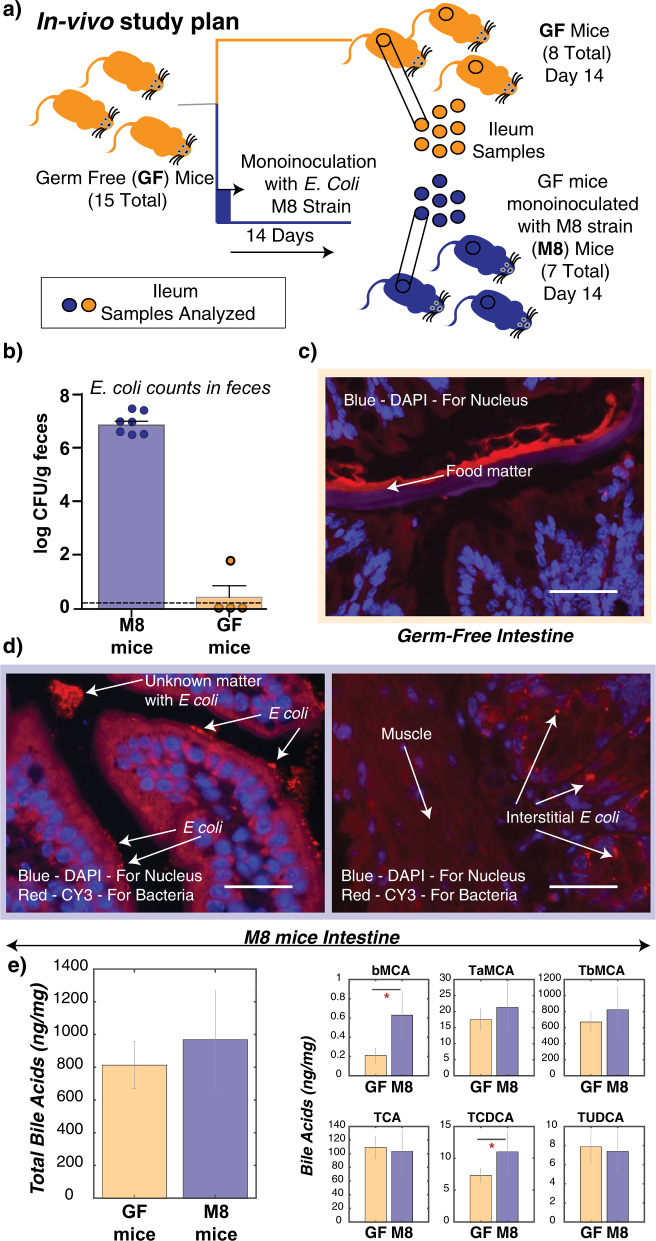
In vivo study plan and findings. **a** In vivo study plan of the monoinoculation experiment. **b** *E. coli* counts in the feces between M8 mice and GF mice feces at the end of the experiment. Bars indicate mean and error bars indicate standard deviation around the mean. **c** FISH analysis of GF ileum slices. No bacteria were observed in the GF cross section. Bar = 50 µm. **d** FISH analysis of M8 mice ileum slices. Bar = 50 µm. *E. coli* was observed in deeper tissue layers including the lamina propria of the mucosa, submucosal spaces and intestinal crypts. **e** Cecal bile acids comparison between GF and M8 mice. Bars indicate mean and error bars indicate standard deviation around the mean. *GF* germ-free, *M8* GF mice monoinoculated with M8 strain of *E. coli*, *DAPI* 4′,6-diamidino-2-phenylindole, *CY3* cyanine 3, *bMCA* β-muricholic acid, *TaMCA* tauro-α-muricholic acid, *TbMCA* tauro-β-muricholic acid, *TCA* taurocholic acid, *TCDCA* taurochenodeoxycholic acid, *TUDCA* tauroursodeoxycholic acid

The microbiome and lipid metabolism are two complex and integrated components that require simplification to identify strategies for potential intervention. We therefore took advantage of gnotobiotic animal models to control for environmental factors including the microbiome composition (Fig. 2a, materials and methods). We decided to focus on the impact of *E. coli* colonization. While *E. coli's* are generally recognized to impact lipid metabolism, there are many strains with diverse characteristics relative to pathogenesis and metabolism. Relevant to our goal of identifying the impact on lipid metabolism, we isolated an *E. coli* strain (M8) from a mouse model of obesity and metabolic disease (Ob/Ob) (previously used in Chakrabarti et al.^27^). Our in vivo studies thus included analysis of germ-free (GF) mice and GF mice inoculated with the M8 strain (referred to as M8 mice) (Fig. 2b, materials and methods). Analysis of the M8 chromosome and plasmid genomes (supplementary S1Data.xlsx) demonstrated the presence of 25 genes relevant for lipoprotein metabolism, including genes for synthesis (*lipA*), trafficking (*lolA*, *lolC*, *lolD*, *lolE* and *lolB*) and lipoprotein export.

In addition to genes directly targeting lipid metabolism, the M8 isolate also contained several genes associated with an adherent and/or invasive phenotype.^28,29^ These included *tibA* and *yadA* adhesion genes previously characterized in Yersinia and ten genes in the colanic acid pathway previously implicated in uropathogenic *E. coli* for adhesion.^30^ Fluorescence in situ hybridization (FISH) analysis demonstrated adherence and invasion of the M8 strain into the mouse ileum consistent with the genomic content of the strain. Tissue-associated M8 were detected in the lumen as well as the lamina propria, submucosal spaces and intestinal crypts (Fig. 2c,d) indicating invasion of the host by the M8 strain. While the observed colonization could be due to opportunistic pathogenesis of an immature GF ileum, the phenotypic characterization of the mouse model was conducive to identify potential pathways by which *E. coli* can regulate lipid metabolism and contribute to the regulation of metabolic health.

Another general property of *E. coli* strains is their bile-acid resistance.^6,31^ In vitro assays demonstrated that this was also the case with the M8 strain (not shown). Since bile plays an important role in absorption of dietary fat, we also examined the bile acid composition of the mice with or without M8 colonization. We observed an overall trend towards increased total bile acids (no statistical difference) upon M8 colonization (Fig. 2e). Six primary bile acids were specifically identified, including TCA (taurocholic acid), TCDCA (statistically higher in M8), TUDCA, bMCA (statistically higher in M8), TaMCA (higher trends in M8) and TbMCA (higher trends in M8) (nomenclatures of bile acids tabulated in Table 1, supplementary S2Data.xlsx). TCDCA, a cytotoxic bile acid, levels increased upon M8 colonization while TUDCA levels did not change significantly. The M8 isolate lacks an annotated *bsh* gene consistent with the lack of secondary bile acids observed in the cecal contents.

**Table 1:** Nomenclature used for bile acids

Short form	Full name
TCA	Taurocholic acid
TCDCA	Taurochenodeoxycholic acid
TUDCA	Tauroursodeoxycholic acid
bMCA	β-muricholic acid
TaMCA	tauro-α-muricholic acid
TbMCA	tauro-β-muricholic acid

Overall, the rationale presented for the preclinical model reproduced many aspects (e.g., pathogenesis, bile acid, and lipid metabolism) by which the microbiome (and in particular *E. coli*) could regulate lipid homeostasis in the host. These properties likely impact host lipid metabolism via a complex integration of signals and host/commensal interactions. We next sought to develop a method to strategically focus our attention to pathways that potentially underlie the regulation of lipid metabolism by the microbiome.

### Transcriptomics-driven lipidomics (TDL)

Current lipidomics analysis focused on legacy knowledge (top branch of Fig. 1) has limitations including the space explored of lipid metabolism and/or technical restraints and results could be confounded by the stability of the lipids, sample processing, and detection limits. For example, currently ~100,000 lipid species have been identified and it would be impossible to capture all these species experimentally. Taking account of the recognized role of the ileum in lipid absorption and the observed association of the M8 strain with the tissue, we extracted mRNA from GF and M8 mice for transcriptomics analysis by microarray. We focused on mRNA because of its recognition as an efficient method for characterizing the metabolic state of target tissue/cells allowing us to take a systems level view of the complexities of lipid metabolism and host/microbe interactions. In order to improve the confidence on the biological interpretation of lipidomics data, we developed TDL (materials and methods), wherein using transcriptomics of the target area under investigation, and using lipid, organism and biochemical databases and GEMs to generate a hypothesis allowing us to predict a condition-specific list of potentially altered lipids for further measurement and analysis.

### Ileum transcriptomics

**Fig. 3:**
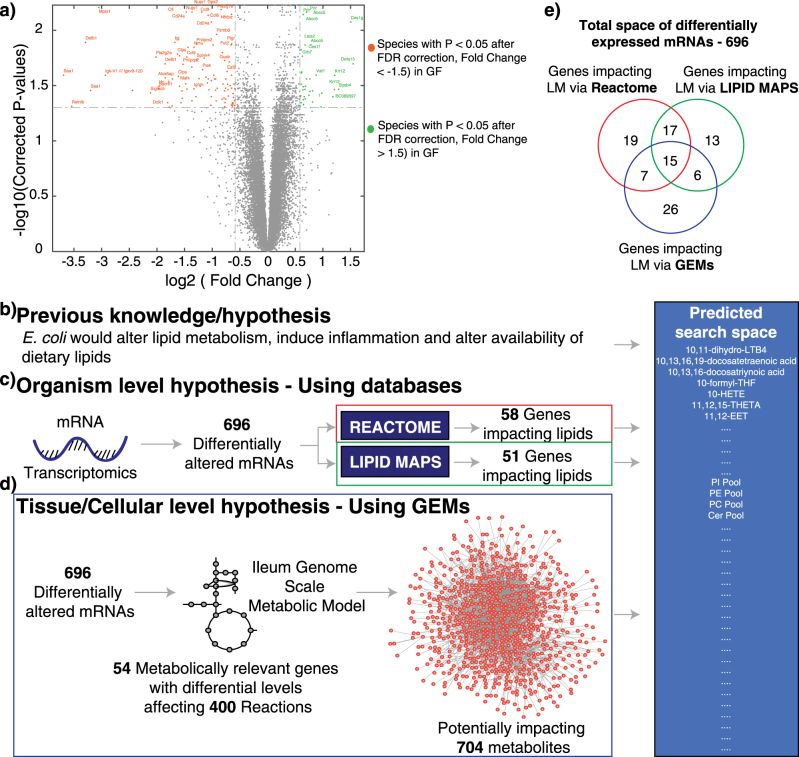
TDL inputs and predictions. **a** Volcano plot of the *P*-values and the fold changes of the ileum transcriptomics data. mRNA species highlighted in green are those with *P*-values <0.05 after FDR correction and with fold change >1.5, i.e., higher in GF. mRNA species highlighted in red are those with *P*-values <0.05 after FDR correction and with fold change <−1.5, i.e., higher in M8 mice. **b** Legacy knowledge about impact of *E. coli* on alterations in lipid metabolism included impacts mediated by inflammation and alterations in dietary lipids. **c** Coupling differential gene expression data from ileum transcriptomics and organism level databases (lipid specific e.g. LIPID MAPS and non-lipid specific, e.g., Reactome) allowed us to predict using different genes potential alterations in lipid metabolism. **d** Coupling differentially expressed genes in the ileum to genome-scale metabolic models (GEMs), we could predict potential alterations in terms of biochemical transformations (reactions) and participating metabolites (including lipids). Compiling the predictions from different branches of TDL, we formulate the predicted search space of altered lipids for further measurement and analysis. **e** Comparison of differentially expressed genes implicated by different aspects of TDL. *GF* germ-free, *M8* GF mice monoinoculated with M8 strain of *E. coli*, *GEMs* genome-scale metabolic models

After corrections for multiple comparisons by FDR, microarray analysis identified 696 differentially expressed mRNA’s (supplementary S3Data.xlsx) between the two groups of mice. 400 transcripts had higher expression levels in GF as compared to 296 higher in M8 mice (Fig. 3a). Strongest upregulated transcripts in M8 mice included *Saa1* (serum amyloid A1), *Retnlb* (resistin like beta), *Mptx1* (mucosal pentraxin 1) and *Defb1* (defensin beta 1) while strongest downregulated transcripts in M8 mice included *Defa15* (defensin alpha 15), *Ces1g* (carboxylesterase 1G) and *Krt12* (keratin 12). In general the microarray data indicated alterations in lipid metabolism, G protein-coupled receptor signaling, immune system signaling, cytokine signaling, Wnt signaling and transmembrane transport upon M8 colonization. In particular *Ces1g* deficiency has been associated with weight gain, insulin resistance, fatty liver and hyperlipidemia through upregulation of de novo lipogenesis and oversecretion of triacylglycerol-rich lipoprotein.^32^ With respect to altered bile acid levels identified earlier, four key genes were differentially expressed between GF and M8 mice in the ileum. These included *SLC2A9*, *Acox2*, *SLC13A2* (all statistically reduced in M8 mice) and *SLC2A10* (statistically increased in M8 mice). These could potentially impact the resorption/circulation of bile acid in the ileum (supplementary S4Data.xlsx). Additionally, further qPCR analysis of *CYP7A1* (statistically higher levels in M8 mice with *P*-values 0.0003 using Mann Whitney test), *CYP27A1*, *CYP7B1* and *CYP8B1* genes in the liver indicated M8 mediated impact in bile acid production in the liver (data not shown).

These data demonstrate that M8 colonization of mice targeted pathways known to regulate lipid metabolism. We next sought to determine how these changes in mRNA reflected the new state of lipid metabolism in M8 mice.

### TDL–transcriptomics and LIPID MAPS

In TDL, we first mapped the differentially expressed genes to the LIPID MAPS Proteome Database (LMPD)^13^ (materials and methods). LMPD with its comprehensive tabulation of major lipid species, regulatory genes and biochemical pathway mappings allowed identification of potential impact of the differentially expressed components specific to lipid metabolism. For *Mus musculus*, of the 1082 unique genes implicated in altering lipid metabolism in LMPD, overlaying differentially expressed genes (696 genes identified above), we identified 51 unique genes potentially affecting lipid metabolism (Fig. 3c and supplementary S5Data.xlsx). Potential effect space of these 51 lipid related genes were analyzed using KEGG^33,34^ and Reactome.^15,16^ Overall, these genes were predicted to impact acyl chain remodeling as well as biosynthesis of glycerophospholipids including PE, PI, PS, PG, PC (abbreviations in Table 2), arachidonic acid and alpha-linolenic acid metabolism, digestion of dietary lipids, glycosphingolipid metabolism, lipoprotein metabolism, very long-chain fatty acyl-CoAs biosynthesis, cholesterol biosynthesis and sphingolipid metabolism.

**Table 2:** Nomenclature used for lipids

Short form	Full name
PE	Phosphatidylethanolamine
PI	Phosphatidylinositol
PS	Phosphatidylserine
PG	Phosphatidylglycerol
PC	Phosphatidylcholine
PA	Phosphatidic Acids
Cer	Ceramide
DAG	Diacylglycerol
TAG	Triacylglyceride
PS	Phosphatidylserine
LPI	Lysophosphatidylinositol
LPC	Lysophosphatidylcholine
LPG	Lysophosphatidylglycerol
LPS	Lysophosphatidylserine
SM	Sphingomyelin
LPA	Lysophosphatidic acids
11-HETE	(±)11-hydroxy-5Z,8Z,12E,14Z-eicosatetraenoic acid
13-HDoHE	13-hydroxy-4Z,7Z,10Z,14E,16Z,19Z-docosahexaenoic acid
13-oxoOD	13-oxo-octadecanoic acid
14-HDoHE	(±)−14-hydroxy-4Z,7Z,10Z,12E,16Z,19Z-docosahexaenoic acid
15-HEPE	(±)−15-hydroxy-5Z,8Z,11Z,13E,17Z-eicosapentaenoic acid
15-HETE	15-Hydroxyeicosatetraenoic acid
17-HDoHE	(4Z,7Z,10Z,13Z,15E,19Z)-17-hydroxydocosa-4,7,10,13,15,19-hexaenoic acid
5-HETE	5-Hydroxyicosatetraenoic acid
LTB4	Leukotriene B4
PGJ2	Prostaglandin J2

### TDL–transcriptomics and reactome

Subsequently, as opposed to constraining ourselves with only pre-curated lipid metabolism-related genes (as discussed above), we explored the potential effect of all the 696 differentially observed genes using Reactome.^15,16^ Specifically for *Mus musculus*, these 696 genes were potentially impacting 789 predicted classes/groups (supplementary S6Data.xlsx). We further shortlisted the predicted list to those affecting lipid metabolism, thus leaving us with 58 unique differentially expressed genes (Fig. 3c) impacting lipid metabolism across 43 different classes/groups. Overall, acyl chain remodeling as well as biosynthesis of glycerophospholipids (PE, PI, PS, PG, PC), phospholipid, glycosphingolipid and sphingolipid de novo biosynthesis, arachidonic acid and alpha-linolenic acid metabolism, cholesterol biosynthesis, triglyceride biosynthesis and lipoprotein metabolism were predicted to be impacted.

Although there were overlaps between LMPD and Reactome in terms of predicted alterations in lipids, exact tissue/cellular specific information about what might be affected and which exact lipid species was still missing. To address this, we next used GEMs of ileum.

### TDL–transcriptomics and GEMs

We used the reconstructed GEM for mouse ileum metabolism^23^ for our current study. Ileum GEM covers about 1353 genes and 4525 reactions involving 3874 metabolites. Essentially, this captures a subset of metabolic enzymes, at the tissue level, giving higher resolution and tissue-specific information about which subset of metabolic enzymes and pathways are relevant in altering lipids. A key point to note is that the GEM used has limitations in terms of depth of granularity for different lipids. Of the 696 differentially regulated transcripts, 54 were relevant/implicated to the ileum GEM (supplementary S7Data.xlsx) (Fig. 3d). These genes could impact reactions in different forms. Some reactions could be directly impacted by differential expression of one gene/reaction (different reactions different genes, one gene per reaction), while others by combinations of two or more genes. For example, in the case of reaction H^+^_[c]_ + NADPH_[c]_ + O_2[c]_ + retinoate_[c]_ = >18-hydroxy-all-trans-retinoate_[c]_ + H_2_O_[c]_ + NADP^+^_[c]_, there are two genes, which could impact it (*CYP2C55* and *CYP4B1*, both differentially expressed). Thus, by translating the statistically different and metabolically relevant genes to the reactions, we predicted that the 54 genes could impact 400 metabolic reactions (supplementary S7Data.xlsx). Of these reactions, 330 were primarily affected by differential expression of one gene. For example, *ACSL6* gene was predicted to impact 114 reactions singularly. For the other reactions (70), there were two or more genes that affected them. These 400 reactions could correspondingly impact directly the levels of 743 metabolites. These metabolites were spread across multiple compartments (e.g., cytosol, mitochondria) and ranged from H^+^ to complex lipids. We filtered this list of metabolites by using the composition filter of C_4_H_8_O_2_ (except for CHO, as a cutoff for lipid and lipid related species) to further narrow down the predicted space of metabolites.

### TDL–formulating consensus predicted lipidome

**Fig. 4:**
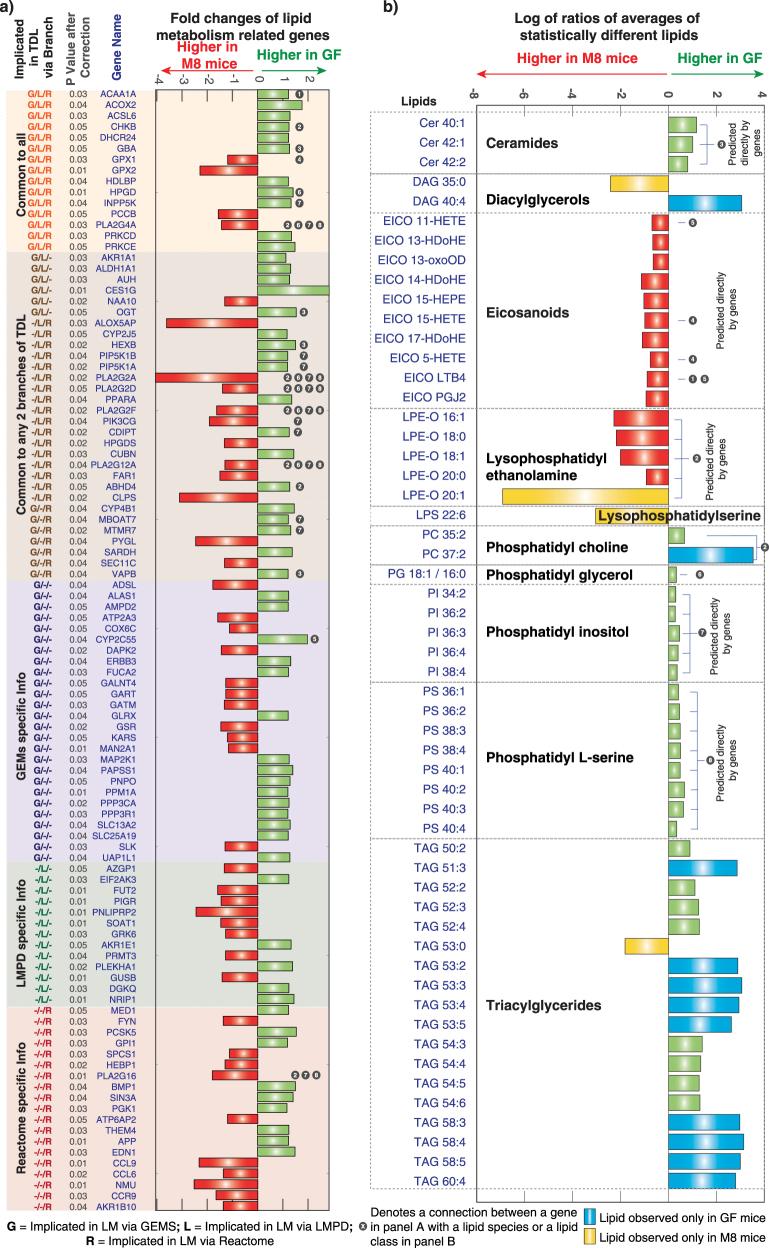
TDL predictions and lipidomics measurements. **a** Fold changes of statistically different genes between GF and M8 mice ileums which are implicated in affecting lipid metabolism in the ileum. Genes are sorted based on their implications in different aspects of TDL. First column indicates whether a corresponding gene is implicated in impacting lipid metabolism via GEMs (G – if yes, else -), via LMPD (L – if yes, else -) and via Reactome (R – if yes, else -). Second column indicates the corrected p values after FDR correction. Third column indicates the gene name. Green filled bars indicate higher expression in GF, while red filled bars indicate higher expression levels in M8 mice ileum. **b** Logarithm of the ratios of the average levels of statistically different lipids between GF and M8 mice ileums are shown. Green filled bars indicate higher lipid levels in GF, while red filled bars indicate higher levels in M8 mice ileum. Blue filled bars indicate lipids observed only in GF samples and yellow filled bars indicate lipids observed only in M8 mice samples. Lipids are sorted based on type. Gray circles with numbers across panel **a** and panel **b** illustrate which gene tabulated in panel **a** is predictive of which lipid species or lipid classes shown in panel **b**. For example, gene *ACAA1* marked with gray circle with the number 1 is predictive of alterations in the levels of the lipid LTB4. *GEMs* genome-scale metabolic models, *LMPD* LIPID MAPS, remaining lipids are tabulated in Table 2

Legacy knowledge and our preclinical results presented above suggested that *E. coli* colonization could alter bioavailability and digestion of dietary lipids, induce inflammatory and immune response and directly contribute to measured lipids and lipid metabolism status in the ileum. Using different branches of TDL we predicted impacts in the level of both lipid species and classes. Owing to inherent differences in the databases, the information contained depths and scope of curation, different sets of genes provided different prediction. For example, 32/58 genes implicated by Reactome to impact lipid metabolism were common to the LMPD database (Fig. 3e), of which 15 were common to GEMs. Similarly, 21 genes were commonly implicated between GEMs and LMPD branches and 22 between GEMs and Reactome branches. Interestingly, 26 genes can be uniquely accounted to impact lipid metabolism by GEMs, 13 uniquely by LMPD and 19 uniquely by Reactome. Fold changes of all the genes (impacting lipid metabolism via Reactome, LMPD and GEMs, 103 genes in total) are shown in Fig. 4a. Genes are sorted based on which branch and how many branches of TDL are accounted to predict changes in lipid metabolism. The top 15 genes (i.e., *ACAA1A* to *PRKCE*) are commonly accounted by all branches of TDL and predictive of impacting lipid metabolism. The next 30 genes (i.e., *AKR1A1* to *VAPB*) are accounted by only two branches of TDL (i.e., either Reactome and LMPD, or LMPD and GEMs or Reactome and GEMs). The next 58 genes (i.e., ADSL to AKR1B10) are accounted by only one branch of TDL in predicting changes in lipid metabolism. Thus each of the branches has unique findings to add to the cumulative predicted altered lipidome. Subsequently, we formulated a consensus predicted lipidome as an unbiased compilation of the predictions (Fig. 3b–d) from all branches of TDL. This predicted altered list comprised of exact lipid species, e.g., LTB4, 1-HETE and lipid classes (e.g., PI, PC pools). Additionally, we also included several lipid classes (predicted to be unaffected) for measurement to serve as negative controls. Relationship between the individual genes and the lipids or lipid pathways for each of the branches of TDL is provided in supplementary S12Data.xlsx.

### Ileum lipidomics

We next evaluated the utility of the TDL strategy to help direct our investigation of the impact of *E. coli* on lipid metabolism in a hypothesis-driven manner. In total, we measured and annotated 383 lipid species (materials and methods). Selection of annotated species was based on the cumulative predicted altered lipid species by the TDL strategy (described above). Of the 383 annotated species, 55 lipids were significantly different between GF and M8 mice ileum samples (Fig. 4b, supplementary S8Data.xlsx).

TDL predictions comprised of ~700 components, including 462 unique species (removing duplicate species across different cellular compartments), 138 CoA’s (which we did not measure due to tissue limitations), 13 pools (1-acylglycerol-3P-LD-TG1 pool, 1-acyl-PE pool, 2-lysolecithin pool, ceramide pool, fatty acid-LD-PC pool, fatty acid-LD-PE pool, fatty acid-LD-TG2 pool, fatty acid-retinol pool, glucosylceramide pool, LacCer pool, PC-LD pool, PE-LD pool, PI pool), 23 species which included non-lipid related cofactors and molecules such as ATP, ADP and NADP amongst others. Of the 288 unique lipid species remaining, 30 belonged to the arachidonic acid metabolism (AAM), specifically identified using the GEM branch of TDL (supplementary S9Data.xlsx). The other branches of TDL also predicted alterations in the pathway level for AAM. This demonstrates one key advantage of using TDL, wherein we obtain exact name of the lipid species to measure based on the study-specific transcriptomics profiles. Genes impacting this prediction included *ACSL6*, *CYP2C55*, *GPX1*, *ACAA1A* and *HPGD* (connections between the genes in Fig. 4a and predicted lipids in Fig. 4b shown using gray number-filled circles). For example, alterations in *GPX1* mRNA levels in M8 mice translated to predicted alterations in 15-HETE and 5-HETE lipids. Correspondingly, we measured over 100 bioactive lipid species within arachidonic cascade^11,35^ out of which, ten lipids were statistically different between GF and M8 mice. All the ten lipids (11-HETE, 13-HDoHE, 13-oxoOD, 14-HDoHE, 15-HEPE, 15-HETE, 17-HDoHE, 5-HETE, LTB4 and PGJ2) had higher levels in the M8 mice (Fig. 4b, abbreviations in Table 2). *ACSL6*, *GPX1*, *ACAA1A* and *HPGD* were commonly accounted for by all branches of TDL to impact AAM. However, *CYP2C55* gene was uniquely accounted for by GEM branch of TDL to impact LTB4 and 11-HETE levels involved in AAM. This demonstrates the utility of using a combination of multiple branches in reaching the comprehensive predicted lipidome for measurement and analysis. *E. coli’s* in earlier studies was implicated in altering levels of AAM components,^36^ which was also reported to mediate inflammatory status in the host.^37^

From TDL, we also get information about lipid classes (or pools) to measure. This is due to a combination of lack of detailed mechanistic knowledge available or compiled in GEMs. We measured and analyzed lipids across different classes: (a) classes predicted from all the branches of TDL, (b) predicted by only one branch of TDL, and (c) predicted by none of the branches of TDL.

All branches of TDL predicted impacts on PI, PC, ceramide, glucosylceramide, DAG, TAG and PE pools. PI’s were predicted to be impacted both by the database branches of TDL (via acyl chain remodeling by genes *PLA2G2F, PLA2G16, PLA2G12A, PLA2G2D, MBOAT7, PLA2G2A, PLA2G4A*, synthesis by genes *CDIPT*, PI metabolism by genes *PIP5K1A, INPP5K, PIP5K1B, PIK3CG* and *MTMR7*) and by the GEM branch of TDL (via gene *MTMR7*, coding for myotubularin related protein 7) (Fig. 4b). Of the 18 PI species measured, 5 (PI 34:2, 36:2, 36:3, 36:4 and 38:4 (all reduced in M8 mice)) were significantly different (Fig. 4b). Similarly, impact on PC’s were predicted by all branches of TDL (via acyl chain remodeling by genes *PLA2G2F, PLA2G16, PLA2G12A, PLA2G2D, PLA2G2A, PLA2G4A* and synthesis by gene *CHKB*) and by the GEM branch of TDL (via gene *PLA2G4A* (higher in M8 mice)) (Fig. 4b). 2/17 PC species (PC 35:2 and 37:2 (both reduced in M8 mice)) were significantly different (Fig. 4b). Alterations in Sphingolipid metabolism was predicted by all the branches of TDL. Specifically, the GEM branch of TDL predicted changes in ceramide and glucosylceramide pools. 3/6 ceramide species (Cer 40:1, 42:1 and 42:2, all reduced in M8 mice) were significantly different (Fig. 4b). Key genes mediating this prediction were *GBA* (fold change 1.26, higher in GF), *VAPB* (fold change 1.20, higher in GF) and *OGT* (fold change 1.52, higher in GF). 2/45 DAGs (DAG 35:0 (increased in M8 mice) and DAG 40:4 (reduced in M8 mice)) were significantly different (Fig. 4b). 18/94 TAGs (TAG 50:2, 51:3, 52:2, 52:3, 52:4, 53:0, 53:2, 53:3, 53:4, 53:5, 54:3, 54:4, 54:5, 54:6, 58:3, 58:4, 58:5 and 60:4 (all except TAG 53:0 were lower in M8 mice)) were significantly different. We observed that 0/23 PEO species, 5/9 LPE-O species (LPE-O 16:1, 18:0, 18:1, 20:0, 20:1 (all increased in M8 mice)) and 0/12 LPE species were significantly different. Similar to the PC pool, the genes *PLA2G2F, PLA2G16, PLA2G12A, PLA2G2D, PLA2G2A, PLA2G4A* and *CHKB* were the key genes predicting the changes for PE’s. For PE’s, the prediction was at a higher level and did not have a deeper resolution into the specific sub-groups.

Impacts on certain lipid classes were predicted by only selective branches of TDL, e.g., PS and PG pools. Both PS and PG pools were predicted to be impacted by the database branches and not the GEM branch of TDL. 8/23 PS species (PS 36:1, 36:2, 38:3, 38:4, 40:1, 40:2, 40:3 and 40:4 (all reduced in M8 mice)) were significantly different (Fig. 4b). 1/12 PG species (PG 18:1/16:0 (reduced in M8 mice)), was significantly different. This illustrates differences in terms of predictive capabilities and scope of different branches of TDL.

Lysophposphatidylinositol (LPI), lysophosphatidylcholine (LPC), lysophasphatidylglycerol (LPG), sphingomyelin (SM), lysophosphatic (LPA), were predicted to be unaffected by TDL. As a negative control, we analyzed these species. Consistent with the predictions, levels of 6/6 LPI’s, 4/4 LPC’s, 17/17 LPG’s, 4/4 SM’s, 15/15 LPA’s were not statistically different.

Several lyso phospholipids were also measured including; LPI (0/6 statistically different between GF and M8), LPC (0/4 statistically different), LPE (0/12 statistically different), LPS (1/17 statistically different), LPA (0/15 statistically different), LPCO (0/4 statistically different), LPG (0/17 statistically different).

In some cases the predictions of TDL were wrong. For example, PA’s were predicted to be impacted by the database branches of TDL but not by the GEM branch. However, 12/12 PA’s measured showed no significant differences. Lysophosphatidylserines (LPS) were predicted not to change by any of the branches. However out of 17 LPS species measured, LPS 22:6 was present only in M8 mice (Fig. 4b). In addition, several lipids, including DAG 40:4, PC 37:2, TAGs 51:3, 53:2, 53:3, 53:4, 53:5, 58:3, 58:4, 58:5 and 60:4 were only observed in GF. These molecules could be reduced directly or indirectly by the presence of M8 to below detection limits (blue filled bars in Fig. 4b).

**Fig. 5:**
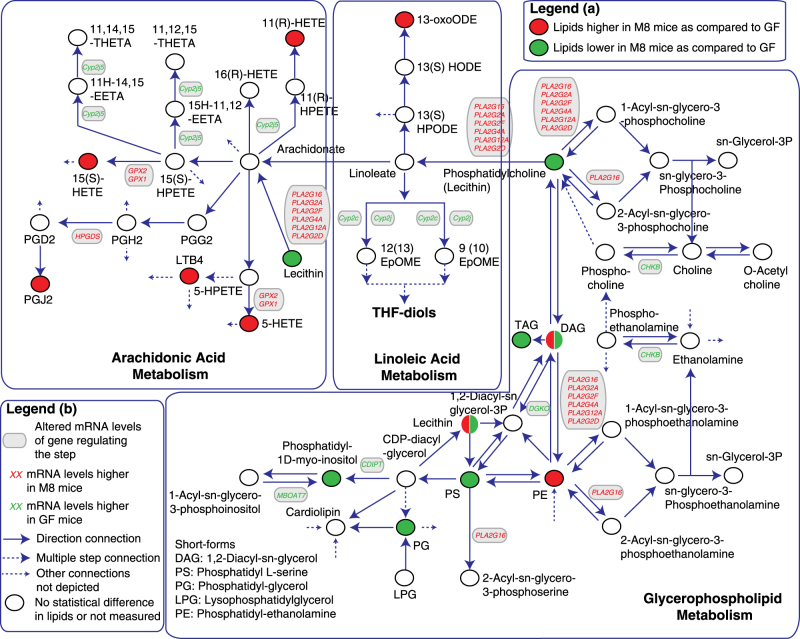
Altered ileum lipid metabolism upon *E. coli* (M8 strain) colonization. Genes indicated in red or green text indicate statistically different fold changes with correspondingly higher or lower amounts in M8 mice as compared to GF mice ileums. Red or green filled circles indicate statistically higher or lower amounts of lipids in M8 mice as compared to GF ileums as demarcated in legend (a). Solid line arrows indicate direct connection between two species, dotted line with arrow between two species indicate multiple step connection and dotted line without a species on one end indicate connections not shown as demarcated in legend part (b)

**Fig. 6:**
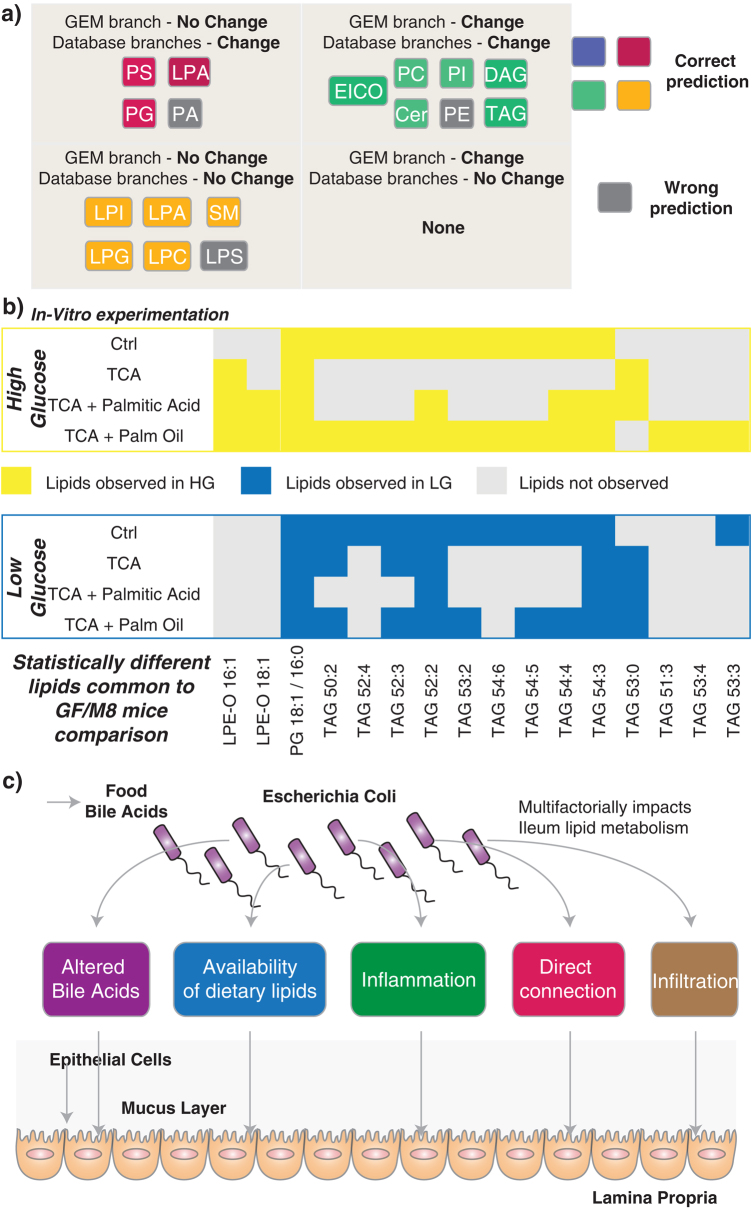
TDL performance and multifactorial impacts on lipid metabolism. **a** TDL performance in predicting changes in different lipid classes. **b** In vitro experiments to identify potential contributions of bacteria to produce the statistically different lipids observed between GF and M8 mice ileum samples. **c** Hypothetical avenues of *E. coli* mediated impacts on ileum lipid metabolism. Abbreviations: lipid abbreviations tabulated in Table 2, *GEM* genome-scale metabolic model, *Ctrl* control, *TCA* taurocholic acid, *HG* high glucose, *LG* low glucose

Overall, the changes in lipid metabolism between GF and M8 mice were focused around a decrease in glycerophospholipids and increase in AAM (Fig. 5). Except for the wrong predictions in case of PA, the presence of LPS 22:6 and lack of resolution in case of PE, TDL predictions were favorably recapitulated in the measured lipids (Fig. 6a, supplementary note). Compiling the predictions of TDL and lipid measurements, we can categorize the different lipid classes into different categories; (a) Category 1: all TDL branches predicted change correctly (e.g., PI, PC, Ceramide pools), (b) Category 2: only one TDL branch predicted change and it was correct (e.g., phosphatidylserine (PS), phosphatidylglycerol (PG) pools), (c) Category 3: all TDL branches predicted no change and it was correct (e.g., LPI, LPC pools), (d) Category 4: only one TDL branch predicted change and it was wrong (e.g., PA pools), (e) Category 5: all TDL branches predicted no change and it was wrong (e.g., PE pool), and (f) where none of the TDL branches predicted change and it was wrong (e.g., LPS pool). Overall, TDL methodology is not perfect and does not give a 100 percent correct prediction. However, as compared to using one branch (i.e., either legacy knowledge, databases or GEMs alone), a comprehensive and integrated strategy, as presented in TDL makes it better poised to provide reliably a predictive altered lipid space for measurement and analysis. Some of the current limitations and improved accuracy can and will eventually be solved as annotations and compilations in databases and GEMs become more encompassing.

Besides the ileum, 127 lipids were measured/annotated/analyzed in the duodenum, 179 in jejunum and 164 in the colon. Data is provided in the supplementary data S11Data.xlsx. There were no statistical differences in the lipids between GF and M8 mice in the jejunum and colon. However, 3/127 lipids, including PGF2a, PG 20:4/18:2 and PI 39:4 (reduced levels in M8 mice) were statistically different between GF and M8 mice in the duodenum. We found a lot of variability in the lipid levels in the duodenum, jejunum and colon as compared to a distinct altered response in the ileum, thus emphasizing our choice to focus on the ileum.

### In vitro assessment of bacterial lipids

One limitation of the metabolic models and databases used is that they can only account for lipid metabolism changes dependent on the state of the host. Interestingly, TAG 53:0, DAG 35:0, LPE-O 20:1 and LPS 22:6 were detected only in M8 samples (yellow filled bars in Fig. 4b). One explanation for the unique presence of these molecules in M8 mice could be that they are potentially of *E. coli* origin. Lipids with odd-chain fatty acids, such as the M8-specific lipids TAG 53:0 and DAG 35:0 are of non-mammalian origin. To investigate the metabolic flexibility and potential direct contribution of M8-derived lipids to the GF and M8 mice profiles, we conducted in vitro studies with the M8 isolate under different growth conditions (low/high glucose, ±Palmitic Acid, ±Palm Oil, ±TCA: materials and methods) (Fig. 6b). A total of 235 lipid species were measured, annotated and analyzed (supplementary S10Data.xlsx). Ninety seven lipids were commonly observed between in vitro and in vivo samples. Maximum diversity of lipids were observed in control and TCA + Palm Oil growth conditions. 16/97 common lipids were observed at statistically different levels between GF and M8 ileums. LPE-O 16:1 and LPE-O 18:1 (both higher in M8) could be selectively produced by M8 in vitro (high glucose + TCA). PG 18:1/16:0 (lower in M8) was produced by M8 irrespective of the in vitro growing conditions. Several TAG’s were detected under specific in vitro growth conditions (Fig. 6b). This included TAG 53:0 that was previously detected only in M8 mice ileums.

Overall, our approach determined five main mechanisms by which host/commensal interactions regulate the lipid composition of the host. These include alterations in bile acid metabolism, the bioavailablility of dietary lipids, inflammatory status, infiltration of commensal bacteria, and bacterial lipids. These mechanisms integrate in a matrix-driven manner and regulate lipid homeostasis in the host. Arachidonic acid and glycerophospholipid metabolism may be potential pathways that can be exploited by microbiome-targeted therapeutics for obesity and metabolic disease.

## Discussion

Metabolic disorders are characterized by altered lipid metabolism, gut microbiota and low-grade inflammation.^38^ Microbiome, by itself is implicated in altering lipid metabolism^39^ and impacting the pathophysiology of several diseases. *E. coli* specifically impacts host lipid metabolism^6^ and is regarded as resistant to the antibacterial functionality of bile acids.^31^ The ileum plays a key role in absorption of luminal bile acids and emulsified dietary lipids.^40^ Thus, modulating the metabolic status of the ileum might be an opportunity for the gut microbes (e.g., *E. coli*) to create an adaptive and facultative local microenvironment. Knowing this, we used a gnotobiotic in vivo system with/without *E. coli* (M8 strain) colonization to investigate lipid metabolism alterations in the ileum to identify molecular mechanisms by which *E. coli* plays a role in obesity and metabolic disorders. In the current study, we focused on the effect of colonization of live *E. coli*. Given the experimental set-up, we cannot predict if the observed lipid changes can occur if GF mice were treated with *E. coli* extracts. Rapid development in lipidomics^8–14,41–45^ have facilitated explorations of lipid metabolism in diverse healthy and diseased states,^4–7,46–53^ primarily driven by legacy knowledge. In this study, we introduce TDL strategy, to address the need of a predictive hypothesis-driven approach to characterize crosstalk between gut microbiota and lipid metabolism. We applied TDL and identified alterations in arachidonic acid cascade and glycerophospholipids as hallmarks of M8 colonization impact on ileum lipid metabolism.

In TDL, we coupled condition-specific systems level measurement of transcriptomics with legacy knowledge, lipid databases (e.g., LIPID MAPS^13^), pathway databases (Reactome^15,16^) and tissue/cellular specific GEMs^23–26^ to hypothesize impacted lipids and pathways following *E. coli* colonization. Thus we leveraged knowledge at different levels of granularity and specificity coupled with mechanistic biochemical transformations and genotype–phenotype information contained in GEMs. Several alternative strategies exist for integration of transcriptomics and GEMs.^25,54–60^ However, they are not tailored to enable hypothesis-driven lipidomics surveys.

Overall, we observed a decrease in glycerophospholipids (e.g., PI, PC, PG, PS, TAGs) and increase in arachidonic acid components (e.g. PGJ2, LTB4, 5-HETE) (Fig. 5). Consistent with our observations, *E. coli* in earlier studies was implicated in altering levels of AAM,^36^ which additionally was reported to impact/mediate inflammatory status in the host.^37^ At the transcriptional level, in the ileum we did not observe any statistical differences between the expression levels of key markers of inflammation like IL6, IL-1b, and TNFa. We observed increase in PGJ2 levels in M8 mice, which previously have been implicated in intestinal inflammation^61^ and IBD.^62^ The gene *PLA2G4A*, encodes a member of the cytosolic phospholipase A2 group IV and catalyzes the hydrolysis of membrane phospholipids to release arachidonic acid, which is subsequently metabolized into eicosanoids.^63,64^ Accordingly, we observed higher *PLA2G4A* expression; lower PC’s and higher AAM components in M8 mice. PCs contribute to the barrier integrity of the gastrointestinal tract. Like PCs, we observed lower levels of several TAGs in M8 mice. It was earlier reported that mono- and diacylglycerol acyltransferases require phosphatidylcholines (PC’s) for optimal activity.^65^ Additionally, we observed higher bMCA and TCDCA cecal bile acids in M8 mice, altered bile acid absorption/circulation related ileum mRNAs and altered bile acid production related mRNAs in the liver, thus demonstrating the recalibration of bile acid metabolism by *E. coli*. This can impact (or be impacted by) PCs,^66^ ceramides^67^ and restructure global ileum lipid metabolism by altering availabilities of dietary lipids in the gut lumen, impacting bacterial lipid metabolism (as seen in the in vitro studies), and/or directly/indirectly altering lipid absorption in the ileum. We also observed lower sphingolipid levels in M8 mice, which are essential structural components of intestinal membranes, providing protection and integrity to the intestinal mucosa and regulating intestinal absorption processes^68^ and plays a key role in immunity and inflammatory disorders, e.g., IBD.^69^ These observations are consistent with altered/reduced ileum integrity in M8 mice, characterized by FISH images of *E. coli* penetrating the ileal villi (Fig. 2d). Linoleic acid and arachidonic acid are also essential for the synthesis of eicosanoids, which are important immune signaling molecules.^70^ The GF state is a sterile condition and inoculation of *E. coli* is expected to trigger an innate and adaptive immune response to the colonization. We observed increase in expression of *Defb1*, which encodes for an antimicrobial peptide. Increase in *Defb1* expression has been reported in IBD.^71^ We also observed increased expression of immune-related mRNAs in the ileum (*Pigr*, *Igj*, *Cfi* and *Ighm)* as well as significantly increased levels of immunoglobulin proteins (IgA, IgG2a, IgG3 and IgM, data not shown) in the plasma of M8 mice. Overall, we hypothesize that alterations in lipid metabolism by *E. coli* colonization are impacted by/impacting the inflammatory and immune status and are therefore potential avenues by which they contribute and reflect *E. coli*’s role in metabolic disease.

Our results demonstrated that hypotheses generated from TDL using transcriptomics data were highly predictable for eicosanoids and several lipid classes. The potential disconnect between transcriptomics and corresponding impacts on enzyme activities have not been investigated in the current study in context of improving/altering predictability of the method. This is definitely an area of improvement and for future studies. Current TDL implementation was limited in its scope to identify lipid metabolism alterations specific to the host. Thus the prediction space could be confirmed experimentally and allowed us to assess the source of other differentially observed lipids not predicted by TDL. These could be derived directly or indirectly by the colonizing microbes. Some of these M8-specific lipids were indeed observed in in vitro studies with *E. coli* under different growth conditions (Fig. 6b). Despite the limited conditions tested, we observed *E. coli* production of TAG 53:0, DAG’s 35:1 and 35:2 in the in vitro studies, suggesting *E. coli* as the potential source of some of the lipids observed specifically in the M8 and suggesting *E. coli*-derived lipids as a potential influence on lipid metabolism in the context of metabolic health. While TAG 53:0 (with odd chain fatty acids, seen in vitro to be produced by M8) is higher in M8 mice; other TAGs (e.g., TAG 51:3, 53:2, 53:3, 53:4) are higher in GF. While these TAGs can be produced by M8 in vitro, they can also originate from other sources such as food. Our observations are a result of metabolism by the host and/or M8 as well as being introduced by food. While we can speculate about the origin of the lipids, we have limited information to confirm the exact route of each lipid species. Further detailed studies would be required to investigate and confirm any hypothesis.

Overall, we coupled a computational strategy to unique analytical methods and their respective data sets to delineate the role of *E. coli* in lipid metabolism in the context of obesity and metabolic health. Specifically, using TDL, we demonstrate the multifactorial nature by which *E. coli* influences lipid metabolism via alterations in bile acids, availability of dietary lipids, inflammation and invasion (Fig. 6c). This systems approach should be relevant to investigate the molecular mechanisms underlying host/microbe interactions as well as other biological areas for investigations.

## Materials and methods

### In vivo experiments

Procedures were approved by “Office Vétérinaire Cantonal du canton de Vaud” Lausanne, Switzerland (Authorization number 2872). All germfree male C57BL/6J mice were purchased at 8 weeks of age from Charles River Laboratories (L’Arbresle, France). Upon arrival, mice were housed individually under a 12-h light/dark cycle for 1 week. All mice were given autoclaved Vittel water (Nestlé Waters, Henniez, Switzerland) and γ-irradiated (40 kGy) chow diet (R03-40, Safe diets, Augy, France). A cohort of seven mice were randomly selected and treated with 10^8^ CFU/mL *E. coli* M8 strain (isolated from the feces of an *ob/ob* mouse) in drinking water for 14 days (Fig. 2a). Fecal *E. coli* quantification was performed by qPCR using specific primers for *E. coli* (Fig. 2b). At the end of the treatment, mice were subjected to an oral lipid tolerance test (with 6 mL/kg of corn oil) after overnight fasting (14 h). The mice were anesthetized by isoflurane 6 h after the lipid tolerance test and tissue samples and cecum content were weighted, flash frozen in liquid nitrogen and stored in a −80 °C freezer for further analysis. Sample size of 4 was estimated using the parameters alpha = 0.05, power = 0.8, effect size = 57% (based on preliminary data in conventional mice study) and two tails. To compensate for the lack of literature information on response of germfree mice to lipid challenge, we used 8 mice as GF control and seven mice for M8 inoculation.

### In vitro experiments

A single *E. coli* M8 strain colony was used to inoculate 5 mL LB broth medium. The pre-culture was allowed to grow for 2–4 h at 37 °C. Then, bacterial solutions were centrifuged for 5 min at 5000 g and the bacterial pellet was re-suspended in a final volume of 10 mL Dulbecco's modified Eagle's medium (DMEM) low glucose (DMEM 1 g/L glucose). Thereafter, 200 µL of the bacterial suspension (~2 × 10^7^ CFU) was added into tubes containing 10 mL of the different growing conditions (TCA, 8 mM; taurodeoxycholic acid, 4 mM; palmitic acid, 250 uM; palm oil, 250 uM) in either high (4.5 g/L) or low (1 g/L) glucose containing DMEM solution. After overnight growth, the solutions were centrifuged for 5 min at 5000 g and washed twice with PBS. The pellets were resuspended in PBS and used for lipidomics analysis.

### Ileum transcriptomics

#### RNA extraction, sample preparation and chip processing

Ileum total RNA was extracted from tissue using RNeasy Mini QIAcube kit (Qiagen, AG, Switzerland), following the manufacturer’s instructions. The quality of RNA samples was checked by using the Fragment Analyzer (Advanced Analytical Technologies, Inc., Ankeny, USA). All cRNA targets were synthesized, labeled, and purified according to the TotalPrep RNA Amplification Kit (Thermo Fisher Scientific, Waltham, MA, USA). This method is based on the Eberwine T7 procedure. Briefly, 300 ng of ileum total RNA were used to produce double-stranded cDNA, followed by in vitro transcription, cRNA labeling with biotin, and fragmentation before hybridizing to the Affymetrix GeneChip mouse genome 430A 2.0 chips (CA, USA), and the results were scanned by an Affymetrix GeneChip scanner 3000 7G.

#### Microarray data processing and statistical analysis

Data were normalized using the robust multichip average (RMA) method. Based on the normal distribution of the data sets, the parametric Pearson’s product moment correlation was applied for quality control. The data matrix was further clustered in order to identify potential outliers. To validate the quality of data sets, a principle component analysis was also applied. One-way analysis of variance (ANOVA) followed by a Benjamini and Hochberg multiple testing correction was applied to discriminate the difference of gene expressions between group A and group B. The corrected *P*-value cutoff was set to 0.05 for further analysis.

### TDL strategy

Often the only metric determining the lipid species to be measured in a biological scenario is legacy knowledge. This approach (top branch of Fig. 1) has its limitations as the space explored for altered lipid state is limited, which in turn maybe relevant or not and is biased with pre-existing knowledge, which might be limited in different disease states. The output however is quantitative and definitive. More often, for the target tissue/cells under investigation, we conduct transcriptomics to obtain a systems level exploration of mRNAs for widespread understanding of the state. This understanding extends to the space of lipids as well. We developed an integrated strategy for hypothesis-driven lipidomics survey coupling transcriptomics and combination of legacy knowledge, lipid databases (e.g., LIPID MAPS^13^), pathway databases (Reactome^15,16^) and tissue specific GEMs^23–26^ to hypothesize predictions of potentially altered lipid metabolism (both at the level of specific lipid species and pathways) in health and disease (Fig. 1). Desired output of TDL is a predicted list, which is a compilation of lipid species and lipid classes, for further measurement and analysis. Formulation of the list consists of different aspects:

#### Legacy knowledge

Often the only metric determining the lipid species to be measured and analyzed in a biological scenario is legacy knowledge. This approach (top branch of Fig. 1) has its limitations as the space explored for altered lipid state is limited, which in turn maybe relevant or not and is biased with pre-existing knowledge, which might be limited in different disease states. Legacy knowledge provides names of exact lipid species to measure along with lipid pathways and depends on the biological state under investigation.

#### Using LIPID MAPS in TDL

LMPD^13^ (downloaded on Aug 5, 2016) provides a comprehensive tabulation of major lipid species with annotations from EntrezGene and UniProt along with relevant information like protein isoforms, orthologs and biochemical pathway mapping for different species. In this database, specifically for *Mus Musculus*, there are 1504 proteins and 1082 genes that are implicated in different ways to alter Lipid Metabolism. For the purposes of TDL, we:

- Map the differentially expressed genes to the genes implicated in LMPD.
- Identify the aspects of lipid metabolism (species and pathways) impacted by the relevant genes.

Output of this branch (obtained by text mining operations, which can be done in excel or with scripts) is a mixture of names of pathways or specific lipids that could be impacted by the differentially observed genes relevant in the scope of lipid metabolism.

#### Using reactome in TDL

Reactome provides a free, open-source, curated and peer reviewed pathway database.^15,16^ For the purposes of TDL, we used Reactome online (www.reactome.org), specifically the analyze data aspect to:

- Upload the differentially expressed genes list for analysis.
- Download and filter the result of the analysis for *Mus musculus*.
- Filter outputs relevant to lipid metabolism.

Output of this branch (obtained by text mining operations, which can be done in excel or with scripts) is a mixture of names of pathways, reaction modules or specific interactions that could be impacted by the differentially observed genes, including those relevant to lipid metabolism.

#### Using GEMs in TDL

GEMs are systems level elucidations of metabolic genotype–phenotype relationships (including that of lipid metabolism) and are valuable tools for investigation of complex, highly interconnected biochemical transformations.^17,18^ With the increase in the number of high quality draft cellular/tissue level GEMs^23–26^ (both for mice and human), it is imperative to take advantage of the metabolic genotype–phenotype relationships in the form of curated cellular/tissue level specific biochemical transformations (including lipid transformations). For the current study, we used the ileum model (mouse specific) published by *Mardinoglu* et al.^23^ For the purposes of TDL, we followed the following steps:

- Read the ileum GEMs using COBRA^72^ toolbox.
- Identify the overlap of the differentially expressed genes and the metabolically relevant genes (information in model.genes) documented in the GEM.
- Identify the reactions (information in model.rxnGeneMat variable) impacted by the GEM relevant (and differentially expressed) genes.
- Identify the metabolites impacted by the above identified reactions, using model.S.

Output of this branch (obtained in the study using MATLAB scripts) is a mixture of specific lipid species and lipid pools along with non-lipid related metabolites.

### Lipidomics analysis

Lipidomics analysis was performed on ileum samples as well as bacterial cell cultures. In summary, approximately 50 mg of ileum tissue was homogenized in 1 mL of ammonium bicarbonate buffer (concentration: 150 mM of ammonium bicarbonate in water) using Tissue Lyser (Qiagen AG, Switzerland) at a speed of 25 Hertz for 2.5 min. A volume of 150 µL of the homogenate was collected for intact lipid analysis, leaving 850 mL for bioactive mediator analysis.

Of 150 µL homogenate, 40 µL was further diluted with 140 µL of ammonium bicarbonate buffer using Hamilton Robot and 810 µL of MTBE /Methanol (7/2 v/v) containing internal standard was added to this mixture. The internal standard mixture contained: lysophasphatidylglycerol (LPG) 17:1, lysophosphatic acid (LPA) 17:0, phosphatidylcholine (PC) 17:0/17:0, PS 17:0/17:0, phosphatidylglycerol (PG) 17:0/17:0, phosphatic acid (PA) 17:0/17:0, LPI 13:0, LPS 13:0, LPC 12:0, lysophosphatidylethanolamine (LPE) cholesterol D6, diacylglycerol (DAG) 17:0/17:0, triacylglycerol (TAG) 17:0/17:0/17:0, ceramide (Cer) 18:1;2/17:0, SM 18:1;2/ 12:0, phosphatidylethanolamine (PE) 17:0/17:0, cholesterol ester 20:0, phosphatidylinositol (PI) 16:0/16:0. The solution was mixed at 700 rpm, 15 min at 4 °C using a ThermoMixer C (Eppendorf AG, Hamburg, Germany) and then centrifugated at 3000 g for 5 min. A volume of 100 µL of the organic phase was transferred to a 96-well plate, dried in a speed vacuum concentrator. Lipid extract was reconstituted in 40 µL of 7.5 mM ammonium acetate in chloroform/methanol/propanol (1:2:4, V/V/V). All liquid handling steps were performed using Hamilton STAR robotic platform with the Anti Droplet Control feature for organic solvents pipetting as described previously.^73^

The remaining 850 µL homogenate was used for bioactive mediator analysis. A volume of 150 µL of 100% methanol was added to the remaining homogenate to bring the volume to 1 mL and spun at approximately 25000 g (5430 R centrifuge, FA-45-24-11-HS rotor) (Eppendorf AG, Hamburg, Germany) for 5 min at 4 °C. Supernatant was removed into new glass tube on ice. One milliliter of 15% methanol was added to pellet and homogenized in Tissue Lyser (25 Hz, 2.5 min). Homogenate was spun (25000 g, 5 min, 4 °C.) and supernatant was added to the glass tube. One milliliter of 15% methanol was used to make a final volume of 3 mL.

Extraction of lipid mediators from the gut tissue was performed according to our published protocol^11^ with slight modifications outlined as follows: Internal standard PGB_2_-*d*4 (40 ng), 12-HETE-*d8* and AEA-*d8* (Cayman Chemicals, Ann Arbor, MI, USA) were added to the homogenized tissue in 15% (v/v) methanol in water. The cartridges (Strata-X 33 u Polymeric Reversed phase 60 mg/3 mL) were washed with methanol (3 mL) followed by water (3 mL) prior to loading the homogenate (3 mL); the cartridges were then washed with 15% methanol in water (3 mL) and lipid mediators were eluted in methanol (3 mL) and collected in glass tubes. The organic solvent was evaporated using a fine stream of nitrogen and the remaining residue was re-dissolved in ethanol (100 µL) and stored at –20 °C awaiting analysis. Lipidomics analysis of intact lipids was performed using QExactive mass spectrometer (Thermo Fisher Scientific) equipped with a TriVersa NanoMate ion source (Advion Biosciences) as described previously.^73^ The data was acquired in both positive and negative mode using resolving power of 140,000 in full scan and 17500 in MS/MS mode. Scan *m/z* range from 200 to 1000. Lipidomics analysis of bioactive lipid mediators was performed as previously described.^35^ Absence of a lipid in any sample was substituted by a value of 0.0001 for calculation of ratios for plotting purposes.

### Bile acid analysis

#### Chemical materials and standard solutions

LC−MS grade organic solvents such as acetonitrile, methanol, water, and formic acid as well as ammonium formate were obtained from Biosolve Chimie (Dieuze, France) or Merck (Darmstadt, Germany). Bile acid standards including 13 deuterium (d4 or d5)-labeled (internal) standards were purchased from either Steraloids (Newport, RI), Toronto Research Chemicals (Toronto, Ontario, Canada) or Medical Isotopes (Pelham, NH 03076, USA). Standards were prepared in stock at 1 mg/mL in methanol and were mixed at 2.5 to 200 μg/mL for bile acids or at 0.25 to 10 μg/mL for 13 D4- or D5-labeled bile acids when being used as internal standards.

#### Sample preparation

A volume of 1500 µL of MeOH:H2O (2:1) with 0.1% (v/v) FA was added to 100 mg lyophilized feces and the samples were homogenized with ceramic beads, in the Cryolys Precellys 24 sample Homogenizer (2 × 20 sec at 10000 rpm, Bertin Technologies, Rockville, MD, USA). Homogenates were centrifuged for 15 min at 4000 g at 4 °C and the supernatant was removed and stored at −80 °C. Prior to solid phase extraction 50 μL of faece extracts were mixed with 100 μL of the ice-cold internal standard solution (in 100% MeOH), and 600 μL of H_2_O with 0.2% (v/v) formic acid in a 2 mL 96-deepwell plates (Waters, Milford, Massachusetts). All plates were heat sealed and mixed by vortexing for 5 min using an Orbit P2 at 1400 rpm (Labnet, Edison, NJ, USA), and centrifuged for 15 min at 4000 g at 4 °C. The mixtures (650 μL of supernatants) were loaded onto an Oasis HLB uElution plate (Waters, Milford, Massachusetts, USA) and the samples was activated with 200 μL of methanol and conditioned with 200 μL of water using the Positive Pressure 96 manifold (Waters, Milford, Massachusetts, USA). Then, the plate was washed with 200 μL of 5% MeOH (v/w) in water under a 3-psi positive pressure, and the analytes were eluted with 100 μL of methanol after 1 min of incubation. The eluates were collected in a Sample recovery collecting plate (350 µL, Waters) and dried under a gentle nitrogen stream at room temperature using a TurboVap 97 (Biotage, Uppsala, Sweden). The dry extracts were reconstituted in 100 μL of 30% acetonitrile in water (v/v). A volume of 20 μL was injected for UPLC−HRMS analyses.

#### UPLC−MS profiling

Bile acids were separated with the reversed-phase chromatographic method with the Acquity UPLC^®^ HSS T3 1.8 µm 2.1 × 100 mm column and following mobile phase system: (a) 5 mM ammonium acetate + 0.1% (v/v) formic acid in water and (b) 0.1% (v/v) formic acid in acetonitrile with a Thermo Accela 1250 UPLC pump and CTC PAL Analytics autosampler (Zwingen, Switzerland) for ultra-performance liquid chromatography (UPLC) at an operating temperature of 40 °C. Quantification of bile acids was performed with a QExactive Hybrid Quadrupole-Orbitrap mass spectrometer (ThermoFisher Scientific, Massachusetts, USA). The MS system was equipped with an electrospray ionization source operating in negative ion mode (ESI−). Mass spectrometry parameters were as follows: full MS 370–522 (centroid acquisition), resolution = 70,000, negative polarity and AGC target = 5e5

#### MS data preprocessing

The LC−MS HR system (QExactive Orbitrap mass spectrometer) is run using XCalibur 2.2 (Thermo Fisher Scientific). The data files were processed into result files using TraceFinder 3.0 (Thermo Fisher Scientific).

### Genome annotation and analysis

The sequencing of the *E. coli* was carried out using the hybrid strategy. First an optical mapping of the genome was performed,^74^ which was followed by a hybrid assembly using a miseq platform with use of shotgun and jump library preparation, the contigs were assembled using spades. The final assembly was performed using the optical mapping data and primer walking for the missing regions. The final finished genome consisted of a single circular genome, a megaplasmid and three smaller plasmids. The annotation of the genome was carried out using BAYsys annotation server^75^ accessed on Dec 2016. The genome consisting of 5.1 MB resulted in 5185 genes being identified and annotated, while the megaplasmid of 1.6 Mb resulted in 224 genes being identified and annotated, the smallest plasmid of 6 kb resulted in a total of 4 genes being annotated. The annotated genes are provided in Supp10Data.xlsx.

### 16S rRNA FISH

Formalin fixed paraffin-embedded sections were deparaffinised, rehydrated, and fixed in 4% paraformaldehyde for 5 min followed by PBS washing. Tissue sections were incubated 10 min/RT in TE buffer containing 10 mg/mL of lysozyme prior to addition of hybridization solution (0.9 M NaCl, 20 mM Tris HCl, pH 8, 0.01% SDS, 30% formamide). Fixed tissue sections were then hybridized with 4.5 ng/µL of a 1⋮1⋮1 molar ratio of the EUB338I, EUB338II, and EUB338III 5′-end-Cy3-labeled 16S rRNA targeted oligonucleotides in hybridization buffer overnight at 35 °C, washed in 65 mM NaCl, 20 mM Tris HCl, pH 8.0, 5 mM EDTA, and 0.01% SDS prior to mounting using dako.

### Statistical tests and analysis

For transcriptomics, ANOVA followed by a Benjamini and Hochberg multiple testing correction was applied to discriminate the difference of gene expressions between GF and M8 mice samples. The corrected *P*-value cutoff was set to 0.05 for further analysis. For lipidomics, statistical difference was assessed using Kolmogorov–Smirnov 2 sample test with FDR correction and accepted as statistically different if Q value was lower than 0.05. For bile acids, statistical difference was assessed using two-tailed, two-sample *T*-tests. Levels of bile acids considered statistically different with *P* values <0.05.

### Data availability

All data generated or analyzed during this study are included in this published article (and its supplementary information files). Specifically, the details are as follows:

Microarray Data: Submitted in GEO (accession number GSE99018), and Supplementary excel sheet S3Data.

Bile acid data: Supplementary excel sheet S2Data.

*E. coli* M8 genome sequence: Presented earlier in *Chakrabarti* et al.^27^ and data in GenBank CP019953-CP019956.

*E. coli* genome annotation: Supplementary excel sheet S1Data.

Lipidomics Data: Supplementary excel sheets S8Data, S9Data and S10Data.

## Electronic supplementary material

SUPPLEMENTARY FILE

Supp_DSMZ report

Data Set 1

Data Set 2

Data Set 3

Data Set 4

Data Set 5

Data Set 6

Data Set 7

Data Set 8

Data Set 9

Data Set 10

Data Set 11

Data Set 12
