# Supplementary material for: Transcriptomics-driven lipidomics (TDL) identifies the microbiome-regulated targets of ileal lipid metabolism
Source: NPJ Syst Biol Appl. 2017 Nov 7;3:33. doi: 10.1038/s41540-017-0033-0 (PMC5676686; doi:10.1038/s41540-017-0033-0)
Supplement: Supplementary file 1 — SUPPLEMENTARY FILE [file 41540_2017_33_MOESM1_ESM.docx]

**Supplementary Materials**

***E. coli* DSMZ characterization**

The *E. coli* used in the current study was first analyzed using polyphasic taxonomy experiments on the isolate. Using API 20E, we found that the bacteria showed positive for beta galactosidase, lysindecarboxylase, ornithindecarboxylase activity. It was also found to produce a positive result for indole production and was able to produce acids by metabolizing several sugar sources including glucose, mannitol, sorbitol, rahmnose, sacchrose, melibiose and arabinose. Using API ZYM, we found that the isolate showed positive and strong activity for alcaline phosphatase, leucin arylamidase, acid phosphatase, beta galactosidase and weak activity for valin arylamidase, trypsin, phosphohydrolase and beta glucosidase. Using API 50 CHE (aerobic and anaerobic), we found that the isolate tested positive for utilization of glycerol, D-arabinose, L-arabinose, ribose, D-xylose, galactose, glucose, fructose, mannose, rahmnose, dulcitol, mannitol, sorbitol, N-acetyl glucosidase, maltose, lactose, melibiose, sucrose, terhalose, raffinose, gentiobiose, L-fucose, and gluconate. Using GEN III OmniLog, we found that the OmniLog database results matched the isolate to species Escherichia coli. The FAME lipid analysis of the bacterial lipids showed a match with *Shigella sonnei* GC subgroup B with a sim index of 0.698, which shares high DNA homology with *E. coli*, this is consistent with FAME based identification for *E. coli* isolates. The total protemics shows a very high homology with *E. coli* with a category match of level A. The closest species identified in the database was the *E. coli* DSM 1576, which is a fecal isolate. Detailed report of the findings are provided in supplementary materials (supplementary Supp_DSMZ_Report.pdf).


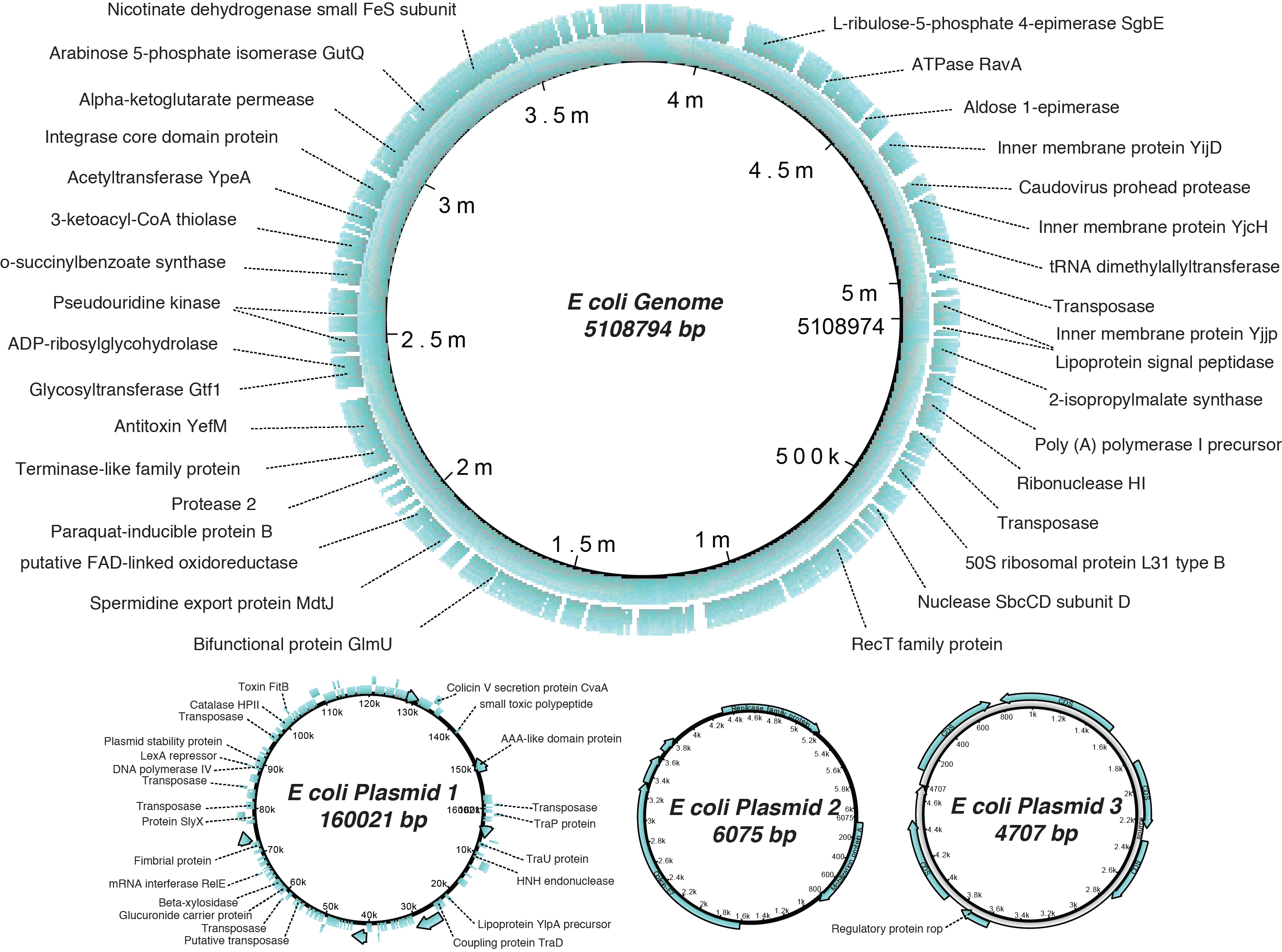


**Supplementary Figure 1**: Visualization of the *E. coli* M8 strain genome.

**Supplementary Figure 2**: Variability of the statistically different lipids between GF and M8 mice. Graphs indicate the mean as the filled boxes with standard deviation shown in the form of errorbars.
