# Supplementary material for: Transcriptomics-driven lipidomics (TDL) identifies the microbiome-regulated targets of ileal lipid metabolism
Source: NPJ Syst Biol Appl. 2017 Nov 7;3:33. doi: 10.1038/s41540-017-0033-0 (PMC5676686; doi:10.1038/s41540-017-0033-0)
Supplement: Supplementary file 2 — Supp_DSMZ report [file 41540_2017_33_MOESM2_ESM.pdf]

Leibniz-Institut DSMZ-Deutsche Sammlung von Mikroorganismen und Zellkulturen GmbH

Leibniz-Institut DSMZ GmbH · Inhoffenstraße 7 B · 38124 Braunschweig · GERMANY

Nestlé Institute of Health Sciences SA
GI Health & microbiome
Attn. Mathieu Membrez
EPFL Innovation Park
Bâtiment H
CH-1015
Lausanne

Inhoffenstraße 7 B
38124 Braunschweig
GERMANY

Tel.: +49(0)531 26 16-0
Fax: +49(0)531 26 16-418
E-mail: contact@dsmz.de
Internet: www.dsmz.de

Ihr Zeichen/Your ref.	Unser Zeichen/Our ref.	+49 (0)531-2616-	Datum/Date
	09014	231<br>identification@dsmz.de	2014-08-15

DSMZ-IDENTIFICATION SERVICE

Dear Sir,

We now have completed the analyses of the lipids and quinones, the phenotypic tests, MALDI TOF and fatty acids for your strain

no. 8 – ID 14-435

Please also check your email account: Mathieu.membrez@rd.nestle.com

Result for the analysis of quinones: Q-7(10%), Q-8 (90%) and MK-8 (100%).

Should the data supplied be used in a publication the following acknowledgement should be included in the "Materials and Methods", "Analysis of respiratory quinones/polar lipids were carried out by the Identification Service, DSMZ, Braunschweig. Germany." Please notice the methods on our webpage.

Where work has been carried out on cell material or strains supplied by the customer, DSMZ makes no guarantee concerning the authenticity of the material/strain supplied.

Yours sincerely,

Leibniz Institute DSMZ-Deutsche Sammlung von
Mikroorganismen und Zellkulturen GmbH

Dr. Susanne Verbarg

Encls. Polar lipids, phenotypic tests, MALDI

Table: API 20 E

**ID-number**	14-435
β-Galactosidase	+
Arginindihydrolase	-
Lysindecarboxylase	+
Ornithindecarboxylase	+
Citrate assimilation	-
H2S-production	-
Urease	-
Tryptophandesaminase	-
Indole-production	+
VP reaction	-
Gelatinase	-
Acid from Glucose	+
Acid from Mannitol	+
Acid from Inositol	-
Acid from Sorbitol	+
Acid from Rhamnose	+
Acid from Saccharose	+
Acid from Melibiose	+
Acid from Amygdalin	-
Acid from Arabinose	+

Leibniz Institute DSMZ – Deutsche Sammlung von

Mikroorganismen und Zellkulturen GmbH

Dr. Susanne Verbarg

2014-08-11

**Table: API ZYM**	
ID-number	**14-435**
Alcaline Phosphatase	4
Esterase	0
Esterase Lipase	0
Lipase	0
Leucin-Arylamidase	4
Valin-Arylamidase	1
Cystin-Arylamidase	0
Trypsin	2
Chymotrypsin	0
Acid Phosphatase	5
Naphtol-AS-BI-Phosphohydrolase	3
α-Galactosidase	0
β-Galactosidase	4
β-Glucuronidase	0
α-Glucosidase	1
β-Glucosidase	0
N-Acetyl-β-Glucosaminidase	0
α-Mannosidase	0
α-Fucosidase	0

Leibniz Institute DSMZ – Deutsche Sammlung von

Mikroorganismen und Zellkulturen GmbH

Susanne Ver

Dr. Susanne Verbarg

Tabelle: API 50 CHE (aerobic and anaerob) 2014-08-11

	API 50 CHE	14-435
1	Glycerol	+
2	Erythritol	-
3	D-Arabinose	+
4	L-Arabinose	+
5	Ribose	+
6	D-Xylose	+
7	L-Xylose	-
8	Adonitol	-
9	βMeDxyloside	-
10	Galactose	+
11	Glucose	+
12	Fructose	+
13	Mannose	+
14	Sorbose	-
15	Rhamnose	+
16	Dulcitol	+
17	Inositol	-
18	Mannitol	+
19	Sorbitol	+
20	αMeDMannoside	-
21	αMeDGlucoside	-
22	N-Acetyl-Glucos.	+
23	Amygdalin	-
24	Arbutin	-
25	Esculin	-
26	Salicin	-
27	Cellobiose	-
28	Maltose	+
29	Lactose	+
30	Melibiose	+
31	Sucrose	+
32	Trehalose	+
33	Inulin	-
34	Melezitose	-
35	Raffinose	+
36	Starch	-
37	Glycogen	-
38	Xylitol	-
39	Gentiobiose	+
40	D-Turanose	-
41	D-Lyxose	-
42	D-Tagatose	-
43	D-Fucose	-
44	L-Fucose	+
45	D-Arabitol	-
46	L-Arabitol	-
47	Gluconate	+
48	2Keto-Gluconate	-
49	5Keto-Gluconate	-

Program	OmniLog 2.3
User	Anja
Data File	IDE_357_140718_A.D5E
Data Location	C:\Program Files\Biolog\OL_DC_23\IDE\Data_2014\07\

Instrument	OmniLog	Project	IDE
Instrument S/N	357	Start Time	Jul 17 2014 2:11 PM
Data Mode	ID	Lapse Time	Jul 18 2014 12:17 PM
Read Mode	Single Read ID	ID Called At	22.00Hrs
Plate Position	11-A	Target Incubation	22 Hrs
Plate Type	GEN III		
Plate Protocol	A		

Sample ID	14-435
Field 2	T=95%
Field 3	n.23h, 28h
Field 4	
Field 5	
Field 6	
Field 7	
Field 8	
Field 9	
Field 10	

Biolog Database	Biolog GEN III 2_6_1_08.I5G
Status	Final ID

Result	Species ID: Escherichia coli
Comment	
Notice	

Rank	SIM	DIST	PROB	Organism Type	Species
1	0.589	5.954	0.589	GN-Ent	Escherichia coli
2	0.183	5.995	0.311	GN-Ent	Citrobacter koseri/youngae
3	0.174	6.044	0.295	GN-Ent	Escherichia coli O157:H7
4	0.035	7.439	0.069	GN-Ent	Shigella sonnei

Key: <x: positive, x: negative, <x-: mismatched positive, x+: mismatched negative, {x: borderline, -x: less than A1 well

Well Color Values

Plate	1	2	3	4	5	6	7	8	9	10	11	12
A	79	{ 129	47 +	{ 149	50	50	< 233	59	60	< 278	< 278	< 285
B	< 223	{ 150	< 191	57	48	{ 143	{ 160	{ 189	< 194	< 273	{ 172	{ 209
C	{ 138	{ 174	{ 163	{ 179	47	72	{ 183	< 199	< 212	< 275	{ 215	98
D	< 204 -	{ 144	48	40	< 218	< 264	< 262	99	49	< 279	< 274	80
E	70	< 223	< 236 -	65	< 237	{ 156	62	64	< 252	< 273	< 255	< 277
F	{ 161	< 249	{ 146	< 250	< 258	{ 124	< 244	68	< 242 -	< 265	< 303	< 317
G	< 209 -	{ 186	< 196 -	< 258	61	68	< 253	< 245	< 250	60	{ 147	83
H	{ 105	72	{ 167	65	{ 143	{ 132	< 198	< 221	83	< 250 -	{ 207	90

Report Date: July 18 2014 12:17 PM (report version: 2.3)

DSMZ Identification Services polar lipid report

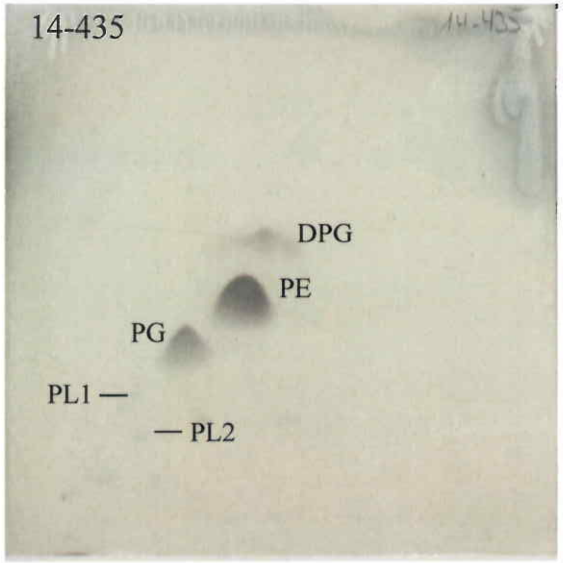

PL = Phospholipid

PE = Phosphatidylethanolamine

PG = Phosphatidylglycerol

DPG = Diphosphatidylglycerol

E147253.30A [30243] UN-V-14-435-N8-LAUSANNE(FS,1d,37C)

Volume: DATA File: E147253.30A Samp Ctr: 6 ID Number: 30243
Type: Samp Bottle: 13 Method: TSBA40 Calc. Method: TSBA6
Created: 7/25/2014 9:56:07 AM
Sample ID: UN-V-14-435-N8-LAUSANNE(FS,1d,37C)

RT	Response	Ar/Ht	RFact	ECL	Peak Name	Percent	Comment1	Comment2
1.571	3.688E+8	0.023	----	7.041	SOLVENT PEAK	----	< min rt	
2.988	426	0.028	----	10.092		----		
3.517	687	0.028	1.105	10.914	Sum In Feature 2	0.29	ECL deviates 0.000	12:0 aldehyde ?
4.421	9538	0.027	1.060	11.999	12:0	3.85	ECL deviates -0.001	Reference -0.002
5.501	386	0.026	1.025	13.001	13:0	0.15	ECL deviates 0.001	Reference -0.002
6.178	1807	0.033	----	13.522		----		
6.801	24358	0.037	0.996	14.001	14:0	9.24	ECL deviates 0.001	Reference -0.003
7.545	1656	0.043	----	14.504	unknown 14.502	----	ECL deviates 0.002	
8.284	3613	0.034	----	15.002	15:0	----	ECL deviates 0.002	
8.570	581	0.035	----	15.179		----		
9.071	20349	0.039	0.962	15.488	Sum In Feature 2	7.46	ECL deviates 0.000	14:0 3OH/16:1 iso I
9.605	9334	0.038	0.956	15.817	Sum In Feature 3	3.40	ECL deviates -0.005	16:1 w7c/16:1 w6c
9.903	98365	0.039	0.952	16.001	16:0	35.69	ECL deviates 0.001	Reference -0.002
10.058	1183	0.053	----	16.092		----		
11.408	68495	0.041	0.938	16.886	17:0 cyclo	24.47	ECL deviates -0.002	
11.600	1540	0.037	0.936	16.999	17:0	0.55	ECL deviates -0.001	Reference -0.001
12.905	971	0.036	----	17.748		----		
13.025	14101	0.045	0.924	17.817	Sum In Feature 8	4.97	ECL deviates -0.006	18:1 w7c
13.336	836	0.039	0.922	17.995	18:0	0.29	ECL deviates -0.005	Reference -0.002
14.099	1287	0.050	----	18.433		----		
14.457	639	0.040	0.914	18.638	19:0 iso	0.22	ECL deviates 0.004	Reference 0.010
14.896	27150	0.044	0.911	18.890	19:0 cyclo w8c	9.43	ECL deviates -0.012	
----	21036	---	----	----	Summed Feature 2	7.75	12:0 aldehyde ?	unknown 10.928
----	-----	---	----	----		----	16:1 iso I/14:0 3OH	14:0 3OH/16:1 iso I
----	9334	---	----	----	Summed Feature 3	3.40	16:1 w7c/16:1 w6c	16:1 w6c/16:1 w7c
----	14101	---	----	----	Summed Feature 8	4.97	18:1 w7c	18:1 w6c

ECL Deviation: 0.004 Reference ECL Shift: 0.004 Number Reference Peaks: 7
Total Response: 282033 Total Named: 275778
Percent Named: 97.78% Total Amount: 262481

Matches:

Library	Sim Index	Entry Name
TSBA6 6.10	0.698	Shigella-sonnei-GC subgroup B (high DNA homology with E. coli)
	0.645	Shigella-sonnei-GC subgroup A (high DNA homology with E. coli)
	0.637	Ewingella-americana
	0.539	Shigella-flexneri (high DNA homology with E. coli)
	0.469	Xenorhabdus-nematophila (48h)

Volume: DATA File: E147253.30A Samp Ctr: 6 ID Number: 30243
Type: Samp Bottle: 13 Method: TSBA40
Created: 7/25/2014 9:56:07 AM
Sample ID: UN-V-14-435-N8-LAUSANNE(FS,1d,37C)

RT	Response	Ar/Ht	RFact	ECL	Peak Name	Percent	Comment1	Comment2
1.571	3.688E+8	0.023	----	7.037	SOLVENT PEAK	----	< min rt	
2.988	426	0.028	----	10.091		----		
3.517	687	0.028	1.107	10.917	Sum In Feature 2	0.28	ECL deviates 0.003	12:0 ALDE ?
4.421	9538	0.027	1.061	12.000	12:0	3.78	ECL deviates 0.000	Reference -0.002
5.501	386	0.026	1.026	13.001	13:0	0.15	ECL deviates 0.001	Reference -0.002
6.178	1807	0.033	----	13.521		----		
6.801	24358	0.037	0.997	14.000	14:0	9.06	ECL deviates 0.000	Reference -0.003
7.545	1656	0.043	0.985	14.502	unknown 14.502	0.61	ECL deviates 0.000	
8.284	3613	0.034	0.974	15.001	15:0	1.31	ECL deviates 0.001	Reference -0.002
8.570	581	0.035	----	15.177		----		
9.071	20349	0.039	0.963	15.487	Sum In Feature 2	7.31	ECL deviates -0.001	14:0 3OH/16:1 ISO I
9.605	9334	0.038	0.957	15.816	Sum In Feature 3	3.33	ECL deviates -0.006	16:1 w7c/15 iso 2OH
9.903	98365	0.039	0.954	16.000	16:0	35.00	ECL deviates 0.000	Reference -0.002
10.058	1183	0.053	----	16.092		----		
11.408	68495	0.041	0.939	16.887	17:0 CYCLO	24.00	ECL deviates -0.001	Reference -0.003
11.600	1540	0.037	0.937	17.000	17:0	0.54	ECL deviates 0.000	Reference -0.001
12.905	971	0.036	----	17.750		----		
13.025	14101	0.045	0.926	17.820	18:1 w7c	4.87	ECL deviates -0.003	
13.336	836	0.039	0.924	17.998	18:0	0.29	ECL deviates -0.002	Reference -0.002
14.099	1287	0.050	----	18.437		----		
14.457	639	0.040	0.916	18.643	19:0 ISO	0.22	ECL deviates 0.009	Reference 0.010
14.896	27150	0.044	0.913	18.896	19:0 CYCLO w8c	9.25	ECL deviates -0.006	Reference -0.005
----	21036	---	----	----	Summed Feature 2	7.60	12:0 ALDE ?	unknown 10.928
----	-----	---	----	----		----	16:1 ISO I/14:0 3OH	14:0 3OH/16:1 ISO I
----	9334	---	----	----	Summed Feature 3	3.33	16:1 w7c/15 iso 2OH	15:0 ISO 2OH/16:1w7c

ECL Deviation: 0.003 Reference ECL Shift: 0.004 Number Reference Peaks: 10
Total Response: 287303 Total Named: 281047
Percent Named: 97.82% Total Amount: 268035

Matches:

Library	Sim Index	Entry Name
TSBA40 4.10	0.702	Shigella-sonnei* (high DNA homology with E. coli)
	0.529	Shigella-flexneri* (high DNA homology wih E. coli)
	0.506	Xenorhabdus-nematophilus* (48h)
	0.457	Morganella-morganii

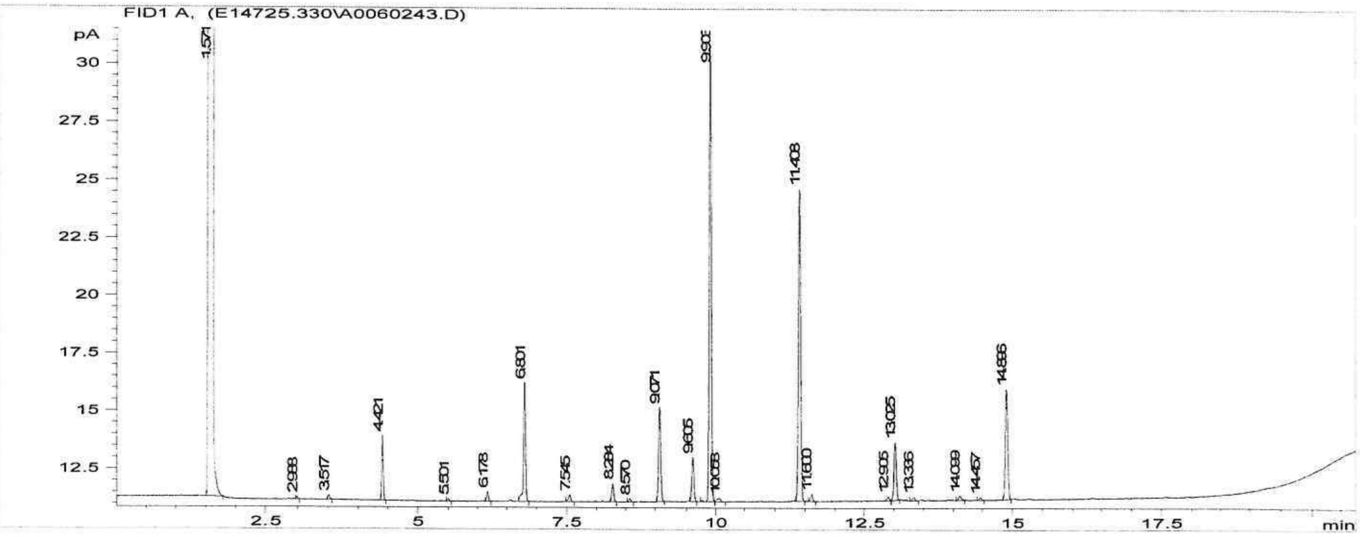
FID1 A, (E14725.330\A0060243.D)
pA
30
27.5
25
22.5
20
17.5
15
12.5
1.571
2.988
3.517
4.421
5.501
6.178
6.801
7.545
8.284
8.570
9.071
9.605
9.903
11.408
11.600
12.905
13.025
13.336
14.099
14.457
14.886
2.5
5
7.5
10
12.5
15
17.5
min

Bruker Daltonik MALDI Biotyper Klassifikationsergebnisse

Projektinformation:

Projektname:	**ID 14-435**
Projektbeschreibung:	Bruker Project Description
Projekt erzeugt von:	psc
Projekt erzeugt am:	11.08.2014 11:01:48
Anzahl der Analyten:	1
Projekttyp:	Development
Validierung:	nicht vorhanden
Validierungsposition:	

Ergebnisübersicht

Analyt-Name	Organismus (bester Treffer)	Bewertungs-zahl	Organismus (zweitbester Treffer)	Bewertungs-zahl
14-435 (+++) (A)	Escherichia coli	2.634	Escherichia coli	2.517

Bedeutung der Bewertungszahlen

Wertebereich	Beschreibung	Symbole	Farbe
2.300 ... 3.000	sehr wahrscheinliche Speziesidentifikation	(+++)	grün
2.000 ... 2.299	sichere Gattungsidentifikation, wahrscheinliche Speziesidentifikation	(++)	grün
1.700 ... 1.999	wahrscheinliche Gattungsidentifikation	(+)	gelb
0.000 ... 1.699	unzuverlässige Identifikation	(-)	rot

Bedeutung der Konsistenzkategorien (A - C)

Kategorie	Beschreibung
A	**Spezieskonsistenz:** Das am besten passende Referenzmuster wurde mit 'grün' klassifiziert (siehe oben). Weitere mit 'grün' klassifizierte Muster stimmen in der Spezies mit dem besten Muster überein. Weitere als 'gelb' klassifizierte Muster stimmen zumindest in der Gattung mit dem besten Muster überein.
B	**Gattungskonsistenz:** Das am besten passende Referenzmuster wurde als 'grün' oder 'gelb' klassifiziert (siehe oben). Weitere mit 'grün' oder 'gelb' klassifizierte Muster stimmen zumindest in der Gattung mit dem besten Muster überein. Die Bedingungen für eine Spezieskonsistenz sind nicht erfüllt.
C	**Keine Konsistenz:** Weder Spezies- noch Gattungskonsistenz (Bitte auf synonyme Namen oder Mischungen von Mikroorganismen überprüfen).

Analyte1

Analytname:	14-435
Analytbeschreibung:	MSP
Analyt-ID:	c6e2b83f-e3d2-46b8-a32c-a2bc5d828d82
Analyt erzeugt am:	24.07.2014 16:19:32
Verwendete MSP-Bibliothek(en):	
Verwendeter Taxonomiebaum:	Bruker Taxonomy

Rang	Übereinstimmende Muster	Bewertungs-	NCBI

(Qualität)		zahl	Code
1<br>(+++)	Escherichia coli DSM 1576 DSM	2.634	562
2<br>(+++)	Escherichia coli RV412_A1_2010_06a LBK	2.517	562
3<br>(+++)	Escherichia coli DSM 682 DSM	2.475	562
4<br>(+++)	Escherichia coli MB11464_1 CHB	2.439	562
5<br>(+++)	Escherichia coli Nissl VML	2.388	562
6<br>(+++)	Escherichia coli ATCC 25922 THL	2.376	562
7<br>(++)	Escherichia coli DH5alpha BRL	2.288	562
8<br>(++)	Escherichia coli ESBL_EA_RSS_1528T CHB	2.267	562
9<br>(++)	Escherichia coli ATCC 25922 CHB	2.267	562
10<br>(++)	Escherichia coli B421 UFL	2.258	562
